# Detecting anxiety and depression among people with limited literacy living with chronic low back pain in Nigeria: adaptation and validation of the hospital anxiety and depression scale

**DOI:** 10.1186/s13690-021-00586-4

**Published:** 2021-05-07

**Authors:** Chinonso Nwamaka Igwesi-Chidobe, Rosemary C. Muomah, Isaac Olubunmi Sorinola, Emma Louise Godfrey

**Affiliations:** 1 Department of Medical Rehabilitation, Faculty of Health Sciences and Technology, College of Medicine, University of Nigeria (Enugu Campus), Nsukka, Nigeria; 2 Department of Physiotherapy, School of Population Health Sciences, Faculty of Life Sciences and Medicine, King’s College London, London, UK; 3 Department of Psychological Medicine, College of Medicine, University of Nigeria (Ituku Ozalla), Nsukka, Nigeria; 4 Department of Psychology, Institute of Psychiatry, Psychology and Neuroscience, King’s College London, London, UK

**Keywords:** Anxiety, Depression, Hospital anxiety and depression scale, Chronic low back pain disability, Nigeria

## Abstract

**Background:**

The Hospital Anxiety and Depression Scale (HADS) is one of the most popular measures of anxiety and depression. The original HADS is mostly used in Nigeria precluding people with limited literacy. This study aimed to cross-culturally adapt and psychometrically test the HADS for rural and urban Nigerian Igbo populations with chronic low back pain (CLBP) who have limited literacy.

**Methods:**

The HADS was forward translated, back translated, and appraised. Face and content validity was ensured by pre-testing the translated measure among a convenience sample of twelve rural Nigerian dwellers with CLBP. Reliability utilising Cronbach’s alpha, intraclass correlation coefficient, Bland–Altman plots and minimal detectable change were investigated amongst a convenience sample of 50 people living with CLBP in rural and urban Nigerian communities. Construct validity testing involving correlations between Igbo-HADS and Roland Morris Disability Questionnaire measuring self-reported back pain-specific disability, World Health Organisation Disability Assessment Schedule assessing generic self-reported disability, Fear Avoidance Beliefs Questionnaire measuring fear avoidance beliefs, and eleven-point box scale assessing pain intensity, and exploratory factor analysis (EFA) and confirmatory factor analysis (CFA) among a random sample of 200 adults with CLBP in rural Nigerian communities were conducted.

**Results:**

Idioms and colloquialisms were difficult to adapt. Internal consistency was good (α = 0.78) and acceptable (α = 0.67) for anxiety and depression subscales respectively. Intraclass correlation coefficients were very good (ICC ≃ 0.8) for both subscales. Minimal detectable change was 6.23 and 5.06 for anxiety and depression subscales respectively. The Igbo-HADS and the anxiety subscale had strong correlations (≃ 0.7) with generic self-reported disability; moderate correlations (≃ 0.5–0.6) with pain intensity, self-reported back pain-specific disability, and fear avoidance beliefs. The depression subscale had the lowest correlations (≃ 0.3–0.4) with these outcomes. The EFA produced a two-factor structure with cross-loading of items. The CFA showed poor fit indices for the EFA structure, the original two-factor structure, and one-factor structure.

**Conclusion:**

The HADS may not be suitable for assessing anxiety and depression, or emotional distress in this population due to difficulty achieving cross-cultural equivalence with western idioms; and the expression of emotional distress through somatisation in this culture.

**Supplementary Information:**

The online version contains supplementary material available at 10.1186/s13690-021-00586-4.

## Introduction

Anxiety and depression facilitate the transition of acute low back pain (LBP) to chronic low back pain (CLBP); and are predictors of CLBP disability in both high income [1–4] and lower income [5–9] countries. Anxiety and depression may however have less influence on work-related disability outcomes such as return to work or sick leave in people with CLBP in high income countries [10–12]. A 40% prevalence rate of depression measured with the hospital anxiety and depression scale have been reported among literate patients living with CLBP in an urban Nigerian population [13]. A large cross-sectional survey involving 6752 literate adults, representing 57% of the general Nigerian population, from five of the six geopolitical zones of Nigeria (including Enugu State), found comorbid conditions which included mood disorders that resulted in a 37% reduction in functioning among 16.4% of a population with CLBP [14]. Limited studies have explored the influence of anxiety and depression on CLBP-disability among people with limited literacy in Nigeria who may have one of the greatest burdens of CLBP globally. Qualitative studies which explored the experience of people living with CLBP in populations with limited literacy in rural Nigeria and the practitioners they consulted [15, 16] implicated anxiety and depression in the experience of CLBP. However, their exact contribution to CLBP outcomes in this population is unclear due to lack of culturally appropriate and valid measures of anxiety and depression.

The hospital anxiety and depression scale (HADS) is one of the most commonly used measures for assessing emotional state because it is reported to differentiate symptoms of anxiety and depression from somatic symptoms of physical illness [17] to enable a clear identification of each. It performs well in assessing the symptom severity and caseness of anxiety disorders and depression in somatic, psychiatric and primary care patients, and in the general population [18]. The HADS compares favourably with other instruments of depression, anxiety and emotional distress or negative affectivity in patients living with CLBP [19], other neuromusculoskeletal conditions [20, 21] and the general population [22, 23]. It appears to be the most consistently used measure of depression and anxiety in Africa, particularly Nigeria [24–27]. These studies have used the original English version of the HADS which is self-administered. People with limited literacy in Nigeria will be unable to read and self-complete the original English HADS which might explain why they have been mostly excluded in studies utilising the original HADS in Nigeria. Unfortunately, the greatest burden of LBP in Nigeria is found among rural dwellers, most of whom have limited literacy. This study aimed to cross-culturally adapt the HADS into Nigerian Igbo for interviewer administration among people with limited literacy in Nigeria, and evaluate the adequacy and psychometric properties of the adapted tool for measuring anxiety and depression, or emotional distress in this population.

## Material and methods

### Study designs

Translation, cultural adaptation, test-retest measurements and cross-sectional study of psychometric properties of the HADS were performed.

### Ethical considerations

Ethical approvals were obtained from King’s College London (Ref: BDM/13/14–99) and the University of Nigeria Teaching Hospital (Ref: UNTH/CSA/329/Vol.5). Written permission was obtained from the original developers of the measure. Informed consent was obtained, and screening was conducted prior to data collection for cross-cultural adaptation, test-retest reliability and validity investigations.

### Cross-cultural adaptation process

#### Participants involved in the cross-cultural adaptation

A health psychologist (native Igbo speaker; bilingual in English and Igbo) who had practised for 9 years in Nigeria and three non-clinical translators (one native Igbo speaker who was bilingual in Igbo and English; one native English speaker who was bilingual in English and Igbo; and one English/Igbo linguistic expert) made up the translation team. An expert review committee included two English experts (health psychologist and academic physiotherapist) working in the United Kingdom, and two Igbo experts (clinical psychologist and clinical physiotherapist) working in Nigeria.

Pre-testing of the Igbo-HADS was done with a convenience sample of adults living with CLBP in rural Nigeria who had participated in a previous qualitative study [15]. All 30 participants that had participated in that qualitative study were invited via telephone to participate in the pretesting of the Igbo-HADS. Twelve participants indicated interest and gave details on when and where the measure could be administered.

#### Procedure utilised in the cross-cultural adaptation

**Fig. 1:**
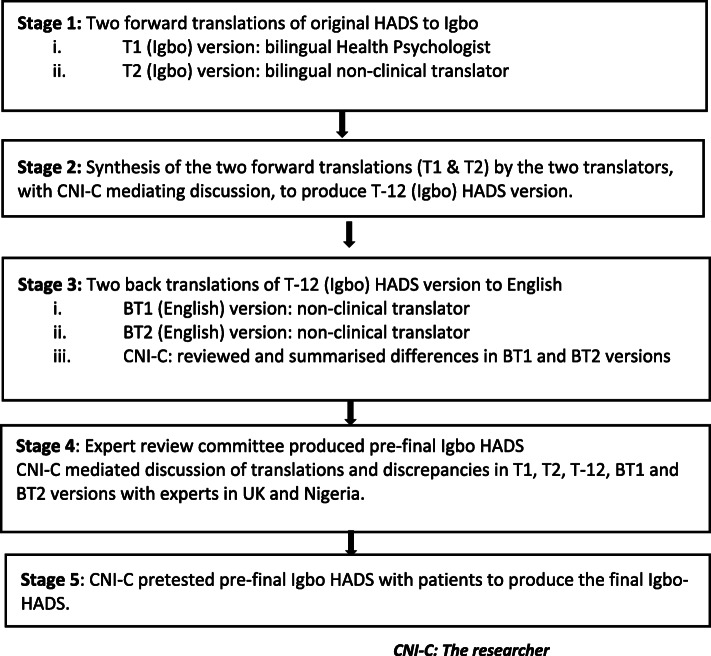
Cross-cultural adaptation stages

Original English version of the HADS was cross-culturally adapted following evidence-based guidelines [28] (Fig. 1).

The questionnaires were forward translated independently from English to Igbo by one bilingual health psychologist and one bilingual translator from a non-clinical background. Both were native Igbo speakers, bilingual in Igbo and English. The items were explained to the health psychologist only. This produced two Igbo versions: T1 and T2 respectively.

T1 and T2 were synthesized via discussion between the two forward translators, mediated by the lead author who is bilingual in English and Igbo. This produced one Igbo version: T-12. Translations were compared and discrepancies were noted.

The Igbo (T-12) versions of the HADS were back translated from Igbo to English by two back translators, blind to the HADS and the construct it measures, who were from non-clinical backgrounds. One of the back translators was an English/Igbo linguistic expert proficient in the professional translation of tools, and the other was a native English speaker, born in England to Nigerian-born Igbo parents. This produced two back-translated English versions: BT1 and BT2. This is a validation process ensuring that the translation was consistent, and that the translated (T-12) versions of the HADS were reflecting the meaning in the original HADS.

T1, T2, T-12, BT1 and BT2 were discussed by the expert committee to produce a pre-final Igbo version of the HADS. The main purpose of this committee was to achieve cross-cultural equivalence in terms of semantic, idiomatic, experiential and conceptual equivalence. For semantic equivalence, the committee explored Igbo and English words to assess if they meant the same thing, if there were multiple meanings to an item, and if there were any grammatical difficulties in the translations. Idiomatic equivalence was assured by the committee formulating alternative Igbo idioms and colloquialisms, where the English versions were difficult to translate. For example, ‘butterflies in the stomach’, an English idiomatic expression for feeling nervous, has a different Igbo equivalent ‘my breathing flying out of my stomach’. Experiential equivalence was achieved by the committee ensuring that questionnaire items were experienced similarly in English and Igbo cultures. For conceptual equivalence, the committee determined that words in the items, instructions, and response options had similar conceptual meanings in Igbo and English cultures. The expert committee also ensured that Igbo wordings were simple and could be easily understood regardless of age and educational levels.

Finally, pre-final Igbo version of the HADS was field tested in rural Nigeria, among twelve participants living with CLBP, who had participated in a qualitative study [15]. This number is above the minimum required sample size of 7 required for the qualitative assessment of the relevance, comprehensiveness and comprehensibility of a translated measure recommended by the Consensus based Standards for the selection of health Measurement INstruments (COSMIN) checklist [29, 30]. The lead author interviewer-administered the HADS using the ‘think-aloud’ cognitive interviewing procedure. Each item was read out, and participants actively verbalised their thoughts as they attempted to answer each question. Participants stated if they encountered difficulty comprehending the items, what was understood by each item, and the meaning of the chosen response. They were encouraged to keep talking while the lead author recorded their responses. This stage ensured that equivalence was achieved in the Nigerian setting to produce the final Igbo-HADS, confirming face and content validity.

### Psychometric testing process

#### Participants involved in the psychometric testing

##### Sampling procedures used to select participants for test-retest reliability

Sample size was determined a priori. A minimum sample size of 27 was required to detect an intra-class correlation coefficient of 0.9 and a maximum width of 0.23 for a 95% confidence interval [31]. For test-retest reliability assessment, a convenience sample of 50 participants with CLBP, between the ages of 18 and 69 years, were recruited from rural and urban communities in Enugu State, South-eastern Nigeria. Community announcements were made in the urban community within which the University of Nigeria Teaching Hospital (UNTH), Enugu is situated and a rural community – Akegbugwu, situated close to it, inviting people with CLBP who were interested in participating in the study to meet at specific community centres. The first 50 people who indicated interest and who met the inclusion criteria at the community centres were recruited for the test-retest reliability assessments.

##### Sampling procedures used to select participants for construct validity investigation

Sample size was also determined a priori. For exploratory and confirmatory factor analysis, a sample size of 200 is deemed sufficient [32]. For correlation analyses, a medium Pearson correlation coefficient of 0.30, at alpha level of 0.05, and 95% power, will require a minimum sample size of 138. Hence, validity assessments were done with a representative random sample of 200 participants living with CLBP in rural communities of Enugu State.

The detailed description of participant sampling and the selection for the cross-sectional validity sample is published elsewhere [33]. A representative sample of the population was ensured through multistage cluster sampling with attempts made to recruit equal number of males and females through gender stratification. Multistage cluster sampling was used to select ten rural communities, representative of rural populations in Enugu State. Twenty households were randomly selected from the list of all households in each community. Data were collected from 20 randomly selected participants from a list of all people with non-specific CLBP in the selected households in each local government area, making a total of 200 participants whose CLBP were not due to underlying serious pathology, radiculopathy or spinal stenosis.

#### Procedures used in the psychometric testing

##### Data quality control for psychometric testing

Fidelity checks were done to avoid systematic differences in data collection. A training manual was produced based on the foundations of good survey design, instructions by the developers of HADS, literature review, and verbal pretesting of Igbo-HADS. Using the manual, ten community health workers (CHWs) were trained for 2 weeks in a classroom at the University of Nigeria Teaching Hospital Enugu, Nigeria, for interviewer-administration of the measures. The CHWs were given post-training assessments, and only those that passed the assessments were recruited as research assistants for data collection. All measures were validated, and the CHWs’ training was tailored to administer the questionnaire items exactly as they were, and to avoid asking questionnaire items in ways that could bias participants’ responses. The CHWs were also trained to ensure that all recruited participants were assessed, and that no items or scales were unanswered.

##### Data collection procedures for psychometric testing

An outcome measure booklet containing screening and demographic questions, and all the listed questionnaires including the HADS was used by each CHW to collect data. Participants were screened first by asking simple questions to rule out back pain associated with underlying serious pathology, radiculopathy or spinal stenosis. They were then requested to describe their pain location with a body chart, before the CHWs interviewer-administered the measures. Likert scales were presented to participants as ‘flash cards’ as each item was read out.

To assess test-retest reliability, the Igbo-HADS was completed at baseline and repeated seven to 10 days after. The same CHW collected data from each participant on the two occasions.

For validity assessment, the Igbo-HADS was completed at one time in a cross-sectional design.

#### Statistical analyses applied in the psychometric testing

IBM SPSS version 22 was used for data analyses. Data were assessed for normality using visual (normal distribution curve and Q-Q plot), and statistical methods (Kolmogorov-Smirnov, Shapiro-Wilk’s test and Skewness/Kurtosis scores).

##### Reliability

Reliability investigates the ability of an instrument to measure consistently [34]. Internal consistency was calculated using Cronbach’s alpha, and was rated as low/weak (0–0.2), moderate (0.3–0.6) and strong (0.7–1.0) [34].

For test-retest reliability, intra-class correlation coefficient (ICC) was calculated using a two-way mixed-effect analysis of variance model with interaction for the absolute agreement between single scores. Random effects model was preferred because of the need to generalize to different raters, and since the retest was performed after a fixed number of days, generalisation to other time points was not required [35]. 0.7, 0.8 and 0.9 represented good, very good and excellent ICCs [36, 37].

Bland-Altman plots were also used to visually assess the level of agreement between test-retest measurements by plotting mean scores against difference in total scores. Bland-Altman analysis accounted for the weakness of ICC which might indicate strong correlations between two measurements with minimal agreement.

Reliability was also evaluated using the standard error of measurement (SEM) and minimal detectable change (MDC). MDC is a statistical estimate of the smallest change detected by a measure that corresponds to a noticeable change in ability which is not due to measurement error. MDC was calculated using the SEM which is based on the distribution method, and the reliability of the measure which takes precision into account). SEM was based on the standard deviation (SD) of the sample and the test-retest reliability (R) of the measure, and was calculated with the equation below [38]:

$$ \mathbf{SEM}=\mathbf{SD}\surd \left(\mathbf{1}-\mathbf{R}\right) $$

MDC was subsequently calculated with the equation below:

$$ \mathbf{MDC}=\mathbf{1.96}\ast \surd \mathbf{2}\ast \mathbf{SEM} $$

### 1.96: 95% confidence interval of no change; √2: because two measurements are involved in measuring change

#### Validity

Validity assesses the extent to which an instrument measures what it is intended to measure. As there are no “gold standard” measures for the Igbo-HADS, construct validity was investigated. Construct validity was investigated using Pearson’s correlation (parametric data) analyses with the Igbo versions of Roland Morris Disability Questionnaire (Igbo-RMDQ), World Health Organisation Disability Assessment Schedule (Igbo-WHODAS), Fear Avoidance Beliefs Questionnaire (Igbo-FABQ) and the eleven-point box scale of pain intensity (BS-11); and were rated as weak (0–0.2), moderate (0.3–0.6), and strong (0.7–1.0) [39].

A priori hypotheses were set. Igbo-HADS is expected to have at least a moderate correlation with Igbo-BS-11 as the literature shows that anxiety and depression are at least moderately correlated with pain intensity [40–42]. Anxiety and depression are also moderately associated with back-pain specific and generic disability, and fear avoidance beliefs [4, 5, 7, 43]. Hence, Igbo-HADS is also expected to have moderate correlations with Igbo-RMDQ, Igbo-WHODAS, and Igbo-FABQ.

### Outcome measures for construct validity investigation

#### Hospital anxiety and depression scale (HADS)

The HADS [17] is a measure of anxiety and depression which have been found to play a key role in the development and maintenance of CLBP. It has two subscales for anxiety (HADS-A) and depression (HADS-D), with seven items each. Each item has scores ranging from 0 to 3. A total subscale score of 0 on either anxiety or depression subscales means there is no anxiety or depression, and 21 is the maximum possible score meaning the most severe anxiety or depression. Summing the scores of anxiety and depression reflects a score of emotional distress with 0 meaning no distress, and 42 meaning highest possible level of emotional distress. Cut-off scores are 0 to 7 for non-cases; 8 to 10 for borderline/mild cases; 11 to 21 for definite/severe cases; with a score of 11 or more indicating “potential psychiatric caseness”. The original measure reported internal consistency of 0.41–0.76 for anxiety, and 0.30–0.60 for depression [17]. Changes of 1.32–1.68 have been reported as clinically important [44].

#### Igbo Roland Morris disability questionnaire (Igbo-RMDQ)

The RMDQ is the most commonly used valid measure of LBP disability [45]. It is a core outcome measure for LBP clinical trials, meta-analyses, cost-effectiveness analyses and multicenter studies. RMDQ is simple to administer, easily understood, and is the best measure for population or primary care-based studies [46]. The Igbo-RMDQ [47] was cross-culturally adapted from the original English version [48]. It is a twenty-four item back specific self-report measure with each item having possible scores of 0 or 1. A total maximum score of 24 denotes the highest possible disability level and 0 means no disability. It has good face and content validity, construct validity, internal consistency, test-retest reliability and responsiveness [49]. The Igbo-RMDQ has Cronbach’s alpha of 0.91; test-retest reliability of 0.84; and a 2–3-point change from baseline is considered clinically important.

#### Igbo World Health Organisation disability assessment schedule (Igbo-WHODAS 2.0)

The WHODAS 2.0 is a comprehensive measure of disability, with an interviewer-administered version that measures disability within the International Classification of Functioning Disability and Health (ICF) biopsychosocial model [50]. It emphasizes all six domains of disability (cognition, mobility, self-care, getting along with people, life activities and participation), and includes work-related disability. Nigeria was one of the 21 countries that contributed data for its development, supporting its cultural sensitivity in Nigeria. As the measure is generic and comprehensive, it would enable comparisons across populations, conditions and an understanding of the disability domains affected. The Igbo-WHODAS 2.0 [51] was adapted from the original English version [52], and has good face and content validity, construct validity, internal consistency, test-retest reliability and responsiveness. It has Cronbach’s alpha ranging between 0.8 and 0.9; test-retest reliability ranging between 0.8 and 0.9; and minimal detectable change ranging between 13.99 and 30.77.

Due to the low literacy levels in this population, the 36-item interviewer-administered version was used using the complex scoring method which takes into consideration multiple levels of difficulty for each WHODAS 2.0 item. This involved summing recoded item scores in each domain, summing all six domain scores, and converting the summary score into a metric ranging from 0 (no disability) to 100 (full disability) [52].

#### Igbo fear avoidance beliefs questionnaire (Igbo-FABQ)

The FABQ [53] is one of the best measures for assessing fear avoidance beliefs. It is a sixteen-item back pain-specific self-report measure that assesses the extent to which pain is believed to be caused or aggravated by general physical activity (FABQ-PA) and work-related activities (FABQ-W). These represent the two subscales of the measure. FABQ-PA has five items, each scored with a Likert scale ranging from 0 (completely disagree) to 6 (completely agree). One item [1] is a distractor and is not scored. The maximum score for FABQ-PA is 24 and the minimum is 0, with higher scores indicating stronger fear avoidance beliefs related to physical activity. FABQ-W has 11 items, each having a Likert scale ranging from 0 (completely disagree) to 6 (completely agree), but four items [8, 13, 14, 16] are distractors, and do not contribute to total score. The maximum score for FABQ-W is 42 and minimum score is 0, with higher scores indicating stronger fear avoidance beliefs related to work activities. Summing the two subscale scores gives a total FABQ score of 64, with higher scores reflecting stronger fear avoidance beliefs. The original FABQ correlates significantly with other measures of fear-avoidance such as the Tampa Scale of Kinesiophobia; r = 0.33–0.59 [54] and a change of 13 from baseline is reported to be clinically important [55]. The Igbo-FABQ was developed from the original English FABQ [56], and has good internal consistency (α = 0.80–0.86); intra class correlation coefficients (ICC = 0.71–0.72); standard error of measurements (3.21–7.40) and minimal detectable change (8.90–20.51).

#### Eleven-point box scale (BS-11)

BS-11 is a single item eleven-point numeric scale for pain intensity. It consists of eleven numbers (0 through 10) surrounded by boxes [57]. Zero represents ‘no pain’ and 10 represents ‘pain as bad as you can imagine’ or ‘worst pain imaginable’. It has good psychometric properties including high test–retest reliability in both literate and illiterate patients with rheumatoid arthritis (ICC = 0.96 and 0.95). It was easier to comprehend and administer than the visual analogue scale (VAS) in this population [15, 33]. The measure is highly correlated (0.86–0.95) with the VAS in patients with rheumatic and other chronic pain conditions; and a reduction of 2 points is regarded as clinically important [58].

Exploratory factor analyses (EFA) was used to determine the number of factors influencing the Igbo-HADS, i.e. the dimensionality of the Igbo-HADS [32]. EFA was applied according to Kaiser Meyer Olkin (KMO) and the Bartlett’s test with a minimum eigenvalue for retention set at ⩾1.0 (Kaiser’s rule) [59]. Retained and excluded factors were also explored visually on a Scree plot. Promax (oblique) rotation, which assumes that factors can be related, was done, and factor loadings less than 0.3 were suppressed as recommended [32]. Extraction was done using principal axis factoring. The number of factors and the underlying relationships between the items were then compared with the factor structures of the original measures to enhance an understanding of population characteristics. Furthermore, confirmatory factor analysis was conducted to determine the model fit indices for the observed structure found the EFA in this study; and the two-factor structure found in the original measure, as well as the one-factor structure reported in the literature [60, 61]. Good fit indices were regarded as a Comparative Fit Index (CFI) of ≥0.90; a Tucker-Lewis Index (TLI), Non-Normed Fit Index (NNFI), and Normed Fit Index (NFI) of ≥0.95; Root Mean Square Error of Approximation (RMSEA) and Standardised Root Mean square Residual (SRMR) of < 0.08 [62, 63].

## Results

There were no missing data.

### Cross-cultural adaptation findings

#### Characteristics of the participants involved in the cross-cultural adaptation

The socio-demographic characteristics of the participants that pre-tested the Igbo-HADS is illustrated in Table 1.

**Table 1:** Socio-demographic characteristics of participants from a rural Nigerian community that pre-tested the Igbo-HADS on 7 July 2014

*n* = 12	Frequency	%
Mean age = 45 years (SD10.36)		
Gender		
Male	7	58.33
Female	5	41.67
Main occupation		
Manual workers	7	58.33
Non-manual workers	5	41.67
Religion (Christian denomination)		
Protestant Pentecostal	10	83.33
Catholic	2	16.67
Marital status		
Married	11	91.67
Single	1	8.33
Educational level completed		
Secondary	4	33.33
Primary	3	25.00
None	3	25.00
Tertiary	2	16.67
Literacy (Ability to read and write)		
Illiterate (inability to read and write)	4	33.33
English	6	50.00
English and Igbo	2	16.67

#### Findings from the cross-cultural adaptation process

##### Translation, comprehensibility and cultural equivalence of Igbo-HADS

The HADS was difficult to cross-culturally adapt. Problems were found with the forward translation of idioms and colloquialisms. Back translations showed the deficiencies in the forward translations. Of the seven items for each subscale, only two items for each subscale reflected the original items. These were: depression – ‘I still enjoy the things I used to enjoy’ and ‘I feel cheerful’; anxiety – ‘I get a sort of a frightened feeling as if something awful is about to happen’ and ‘I feel restless as if I have to be on the move’. All the items in the HADS were reviewed again by the expert committee. The Igbo clinical Psychologist (PhD in clinical Psychology), with over 20 years of clinical experience with Igbo patients) in the expert review committee, advised on the use of equivalent Igbo idioms and colloquialisms. Modifications included replacing ‘wound up’ with its Igbo equivalent ‘not relaxed’, and ‘butterflies in the stomach’ with its Igbo equivalent ‘my breathing flying out of my stomach’. Repeat back-translations of the Igbo-HADS showed that all items had achieved conceptual equivalence while retaining cultural sensitivity. During verbal pre-testing, some participants understood the initial translation of ‘restless’ as ‘useless in life’. Hence, ‘I don’t have rest’, understood as ‘restless’, was used instead in combination with ‘I am not able to stay still … ,’ to reflect the original item.

### Psychometric properties

#### Characteristics of the participants involved in the psychometric testing including reliability and validity investigations

The demographic characteristics of the two samples are presented in Tables 2 and 3.

**Table 2:** Demographic characteristics of participants from rural and urban Nigerian communities involved in the test-retest reliability testing of the Igbo-HADS on 11 August 2014

*n* = 50	Frequency (%)	Mean (SD)
**Gender**		
Female	32 (64.0)	
Male	18 (36.0)	
**Habitation**		
Rural	20 (40.0)	
Urban	30 (60.0)	
**Age (years)**		45.2 (11.55)
**Education (years)**		13.3 (7.14)
**Current marital status**		
Currently married	37 (74.0)	
Never married	8 (16.0)	
Widowed	4 (8.0)	
Separated	1 (2.0)	
**Work status**		
Paid work	25 (50.0)	
Self-employed (own business or farming)	19 (38.0)	
Keeping house/homemaker	2 (4.0)	
Student	2 (4.0)	
Non-paid work (volunteer or charity)	1 (2.0)	
Unemployed (health reasons)	1 (2.0)

**Table 3:** Demographic characteristics of participants from rural Nigerian communities involved in the cross-sectional validity testing of the Igbo-HADS on 22 August 2014

*n* = 200	n (%)	Mean (SD)
**Sex**		
Female	112 (56.0)	
Male	88 (44.0)	
**Age (years)**		48.6 (12.0)
**Education (years)**		7.0 (6.4)
**Current marital status**		
Currently married	143 (71.5)	
Widowed	31 (15.5)	
Never married	22 (11.0)	
Cohabiting	2 (1.0)	
Separated	2 (1.0)	
**Work status**		
Self-employed (own business or farming)	125 (62.5)	
Paid work	31 (15.5)	
Non-paid work (volunteer or charity)	16 (8.0)	
Keeping house/homemaker	13 (6.5)	
Student	7 (3.5)	
Unemployed (health reasons)	4 (2.0)	
Unemployed (other reasons)	3 (1.5)	
Retired	1 (0.5)	

#### Reliability findings

**Fig. 2:**
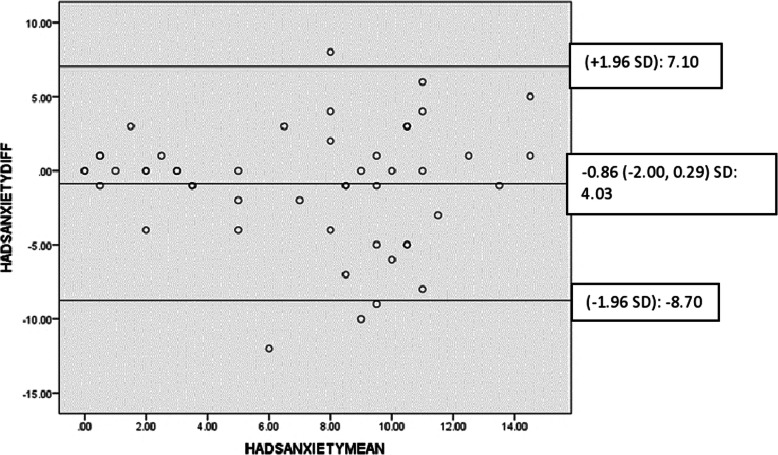
Bland-Altman plot for test-retest agreement of lgbo-HADS (anxiety)

**Fig. 3:**
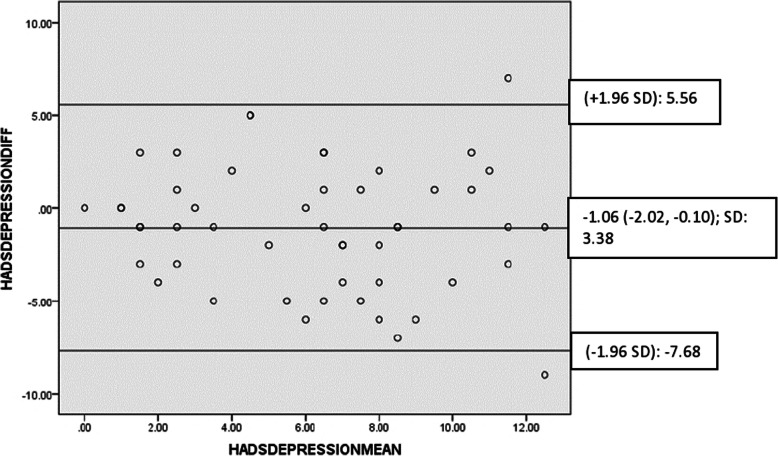
Bland-Altman plot for test-retest agreement of lgbo-HADS (depression)

The test-retest reliability of the Igbo-HADS is illustrated in Table 4. There was acceptable agreement between test-retest values of the anxiety and depression subscales of the Igbo-HADS with mean differences that were close to zero and most points that were within the 95% limits of agreement of the mean differences (Figs. 2 and 3).

**Table 4:** Reliability of Igbo-HADS [obtained from participants from rural and urban Nigerian communities involved in the test-retest reliability testing on 11 August 2014]

**Igbo-HADS anxiety subscale** Number of items: 7; Cronbach’s alpha global score: 0.78; ICC (95% CI): 0.76 (0.58, 0.86)							
Cronbach’s alpha If Item Deleted							
A1	**A3**	**A5**	**A7**	**A9**	**A11**	**A13**	
0.78	0.75	0.75	0.79	0.72	0.75	0.72	
SEM: 2.25 MDC: 6.23							
**Igbo-HADS depression subscale** Number of items: 7; Cronbach’s alpha global score: 0.67; ICC (95% CI): 0.75 (0.55, 0.86)							
Cronbach’s alpha If Item Deleted							
D2	**D4**	**D6**	**D8**	**D10**	**D12**	**D14**	
0.62	0.63	0.60	0.68	0.64	0.59	0.68	
SEM: 1.82 MDC: 5.06							

*ICC* Intraclass correlation coefficient, *SEM* Standard error of measurement, *MDC* Minimal detectable change

#### Construct validity findings

**Fig. 4:**
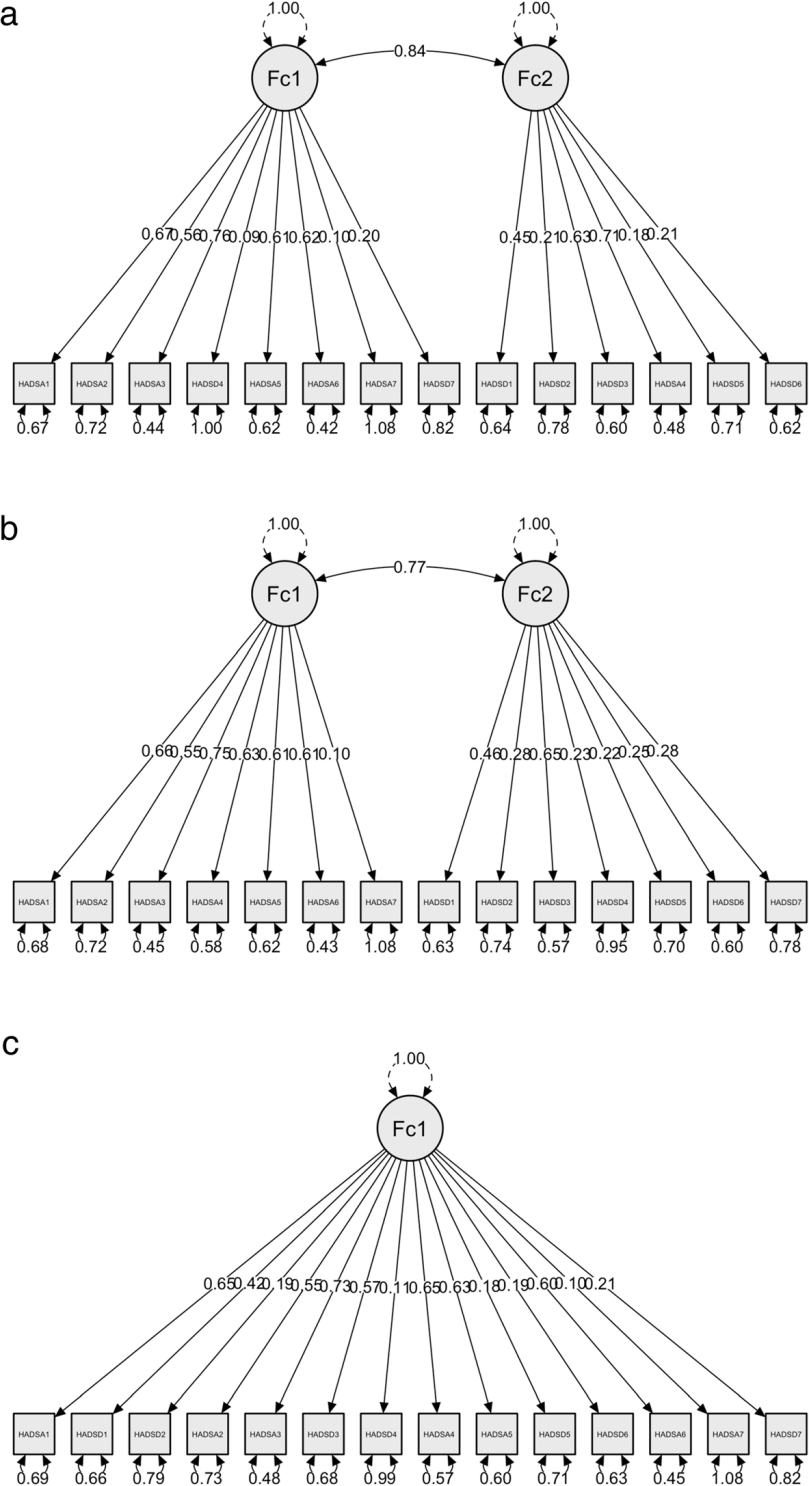
**a** lgbo-HADSS EFA structure applied to CFA. **b** Two-factor structure of original HADS. **c** One-factor structure applied to lgbo-HADS in CFA

The correlations between Igbo-HADS and its subscales with pain intensity (BS-11), back pain specific (Igbo-RMDQ) and generic disability (Igbo-WHODAS), and fear avoidance beliefs (Igbo-FABQ) are shown in Table 5. A two-factor solution of the Igbo-HADS is illustrated in Table 6. 64.29% of the items had factor loadings above 0.5 and 78.57% of the items loaded on their corresponding factor in the original measure: 85.71% for anxiety subscale; 71.43% for depression subscale. Poor fit indices are highlighted (Table 7) with the application of the Igbo-HADS EFA structure to CFA (Fig. 4a), application of the two-factor structure of the original HADS to Igbo-HADS in CFA (Fig. 4b), and application of the one-factor structure to the Igbo-HADS in CFA (Fig. 4c).

**Table 5:** Correlation analyses of the Igbo-HADS [obtained from participants from rural Nigerian communities involved in the cross-sectional validity testing on 22 August 2014]

	Igbo-HADS (anxiety)	Igbo-HADS (depression)	Igbo-HADS (total)
BS-11	0.484**	0.373**	0.503**
Igbo-RMDQ	0.570**	0.300**	0.521**
Igbo-WHODAS (total)	0.685**	0.528**	0.712**
Cognition	0.580**	0.470**	0.614**
Mobility	0.625**	0.403**	0.610**
Self-care	0.494**	0.362**	0.504**
Getting along	0.408**	0.322**	0.428**
Life activities	0.565**	0.389**	0.564**
Participation	0.549**	0.539**	0.629**
Igbo-FABQ total	0.545**	0.380**	0.546**
Igbo-FABQ (PA)	0.492**	0.353**	0.498**
Igbo-FABQ (W)	0.522**	0.353**	0.517**

**p < 0.01 *p < 0.05

**Table 6:** Exploratory factor analysis of the Igbo-HADS [obtained from participants from rural Nigerian communities involved in the cross-sectional validity testing on 22 August 2014]

	1	2
(A2) Something awful	.744	
(A7) Sudden panic	.666	
(A6)Restlessness	.651	
(A3) Worrying thoughts	.628	
(A1) Wound up	.623	
(D4) Lost interest in appearance	.559	
(A5) Butterflies in the stomach	.510	
(D7) Slowed down	.473	
(D2) Funny side of things		.675
(A4) Sit at ease and feel relaxed		.542
(D1) Still enjoy things I used to enjoy		.438
(D6) Enjoy book, radio, tv		.403
(D5) Look forward with enjoyment to things		.392
(D3) Feel cheerful		.378
KMO = 0.82		
Χ^2^ = 617.22***		

Only factor loadings above 0.3 are shown; KMO = Kaiser-Meyer-Olkin measure of sampling adequacy; χ^2^ = Bartlett’s test of sphericity tested with chi-square ***p < 0.001; Extraction Method: Principal Axis Factoring; Rotation Method: Promax with Kaiser Normalization; Rotation converged in 3 iterations.

**Table 7:** Model fit indices for the confirmatory factor analyses of the Igbo-HADS for the observed EFA structure, original two-factor and one-factor structures [obtained from participants from rural Nigerian communities involved in the cross-sectional validity testing on 22 August 2014]

Igbo-HADS CFA applications	Model fit indices						
	X^2^ (*P*-value)	CFI	TLI	RMSEA	SRMR	NNFI	NFI
Igbo-HADS EFA structure	182.608 (< 0.001)	0.805	0.767	0.084	0.089	0.767	0.714
Original HADS two-factor structure applied to Igbo-HADS	180.946 (< 0.001)	0.808	0.770	0.083	0.087	0.770	0.716
One-factor structure applied to Igbo-HADS	193.785 (< 0.001)	0.786	0.748	0.087	0.090	0.748	0.696

*CFA* Confirmatory Factor Analysis, *X*^*2*^ Chi-square, *CFI* Comparative Fit Index, *TLI* Tucker-Lewis Index, *RMSEA* Root Mean Square Error of Approximation, *SRMR* Standardised Root Mean square Residual, *NNFI* Non-Normed Fit Index, *NFI* Normed Fit Index.

## Discussion

### Summary of main findings and their interpretation in relation with current literature

The HADS was difficult to cross-culturally adapt and validate for reasons that may include population characteristics and inherent attributes of the HADS instrument.

The British idioms and colloquialisms were not familiar in this culture in line with reports of other non-English adaptations [64]. An Igbo clinical psychologist familiar with the idioms and colloquialisms commonly used in this culture helped to achieve semantic, idiomatic, experiential, and conceptual equivalence with the Igbo-HADS through cross-cultural adaptation. Subsequent pre-testing of the Igbo-HADS among the people with CLBP improved and confirmed comprehensibility, comprehensiveness and acceptability. Furthermore, qualitative studies (published after this study), suggest that emotional distress may be expressed using physical symptoms in this population. For instance, people described their experience of living with CLBP in rural Nigeria as ‘a life of living death’, explaining their prolonged hopelessness due to CLBP. They also described ‘tiredness’ in relation to depression; ‘escaping from the self’ and feelings of ‘something moving about the body’ in relation to extreme emotional distress [15, 16]. Expression of emotional distress through somatisation is common in other non-western settings [65, 66]. Articulation of emotional distress using somatisation, and the unclear concepts of anxiety and depression in this population [15, 16], may suggest that the HADS which excludes somatic symptoms, and separates anxiety and depressive symptoms may not be the best measure for assessing emotional distress in this population. Another problem could be the illiteracy of the people involved in this study which warranted the adaptation of the Igbo-HADS for interviewer-administration, as opposed to the original measure which was self-administered. Although evidence suggests that interviewer-administration is comparable to self-administration [67], social desirability bias [68] could have been implicated which would mean that participants responded in ways that they felt was acceptable, rather than how they truly felt. However, what is more likely is that limited literacy in this culture that expresses emotional distress predominantly through physical symptoms could have meant that the participants could not understand or relate to the items in the Igbo-HADS.

The inherent shortcomings of the HADS could explain some of the structural validity limitations of the Igbo-HADS found in this study. Ambiguous items, limited breadth and depth of content for anxiety and depression, lack of separation between symptoms of anxiety and depression, and inconsistent factorial structure [60, 61, 69, 70], has led to calls to abandon the use of the HADS [71]. Others have reported an improvement in the structure and validity of the HADS as a unidimensional scale with exclusion of three items and the recoding of one item [72]. However, many studies have reported good fit indices with the original bifactor model and/or a unidimensional model [22, 23, 60, 70, 73] which could not be replicated in the CFA conducted in this study. The poor model fit indices in the CFA of the Igbo-HADS could be due to the characteristics of this population as previously described, which suggest that the HADS might not be the best tool for this population.

The construct validity findings of the Igbo-HADS using the two-factor structure of the original measure appear acceptable. The Igbo-HADS and the anxiety subscale had strong correlations (≃ 0.7) with generic self-reported disability (Igbo-WHODAS); moderate correlations (≃ 0.5–0.6) with pain intensity (BS-11), self-reported back pain-specific disability (Igbo-RMDQ), and fear avoidance beliefs (Igbo-FABQ) which agree with the literature [2–8, 13, 33]. The stronger correlation of the Igbo-HADS with generic self-reported disability than with self-reported back pain-specific disability and other measures could be because of the involvement of cognition and getting along constructs in the generic self-reported disability which closely align with the emotional construct in this population [51]. The depression subscale had the lowest correlations (≃ 0.3–0.4) with these measures possibly because people express emotional distress through somatisation in this population, which appears to be more related to anxiety than depression [74]. This might explain why the factor corresponding to anxiety in the EFA was more consistent with the original measure than depression. There was cross-loading of items in the two-factor solution of the Igbo-HADS in the EFA. Factor 1 corresponds to the anxiety subscale of the original measure except for one missing item (sit at ease and feel relaxed) that loaded on the depression factor, and two items of the depression subscale (slowed down, and lost interest in appearance) that loaded on the anxiety factor. Factor 2 matches the depression subscale of the original measure except for the above cross-loadings. These findings support the unclear separation of the constructs of anxiety and depression found in this population [15, 16] as well as in the HADS [60, 61].

Reliability indices of the Igbo-HADS using the two-factor structure of the original measure also appear adequate. Anxiety and depression subscales of Igbo-HADS had internal consistencies (α = 0.78; α = 0.67) that were in line with the original measure [17]. The lower internal consistency of the depression subscale when compared with the anxiety subscale, is consistently found in other studies [75, 76]. This could be because depression may be a less basic and physiological emotional state than anxiety. Exclusion of somatic symptoms from the HADS, may have further increased inconsistency in this non-western setting where emotional states are often expressed through somatisation [15, 65, 66]. Good reliability of the Igbo-HADS (ICC ≃ 0.8) was demonstrated which agrees with the original measure [17], and adapted versions [77, 78]. Bland-Altman plots showed good agreement between test-retest values. SEM of 1.82 and 2.25, MDC of 5.06 and 6.23, and limits of agreement of − 7.68 to − 8.70, and 5.56 to 7.10 of depression and anxiety subscales of Igbo-HADS, all exceed the minimal clinical important difference of between 0.5 and 5.57 reported in the literature [79].

### Strengths and limitations

This study enabled the investigation of the suitability of the HADS as a measure of emotional distress for non-English speaking Igbo Nigerians with limited literacy. Other strengths of this study include good acceptability of the items in the Igbo-HADS, correlations with generic and back pain specific disability, fear avoidance beliefs and pain intensity in line with established literature.

However, low literacy rates, interviewer-administration in place of self-administration, and data collection by several raters may have increased sample variability and measurement errors which may have influenced the findings in this study. Sensitivity-to-change studies of the Igbo-HADS are required in populations of varying literacy levels (including those that are literate to enable self-administration), with single raters, and using analysis such as receiver operating characteristic (ROC) curves, which includes patients’ own global impression of change. These studies need to confirm the MDCs of the Igbo-HADS and its subscales plus the proportion of people that achieve these MDCs as well as its structural validity. Due to lack of any existing Igbo measure of emotional distress, criterion validity could not be directly investigated. As other Igbo measures of emotional distress become available, they can be used to ascertain the criterion validity of the Igbo-HADS. There was lack of bilingual assessment of the item-by-item agreement of the original and Igbo-HADS as well as a comparison of self-administration with interviewer-administration. This should be investigated in future studies involving populations with adequate literacy levels to enable reading and comprehension of English and Igbo. As cross-cultural adaptation ensures the cultural fit of an instrument beyond simple translation, another limitation of this study is the preservation of the original factor structure of the HADS in the Igbo-HADS in the construct and reliability assessments. Although none of the fit indices in the CFA of the Igbo-HADS were adequate, the original factor structure of the HADS produced the best fit indices when applied to the Igbo-HADS. Future studies need to investigate the structure of the Igbo-HADS using larger sample sizes and varying population group characteristics.

## Conclusions

Cross-cultural equivalence was difficult to achieve, and the psychometric characteristics of the Igbo-HADS suggest that the HADS may not be the best tool for assessing anxiety and depression, or emotional distress in this population with limited literacy and in a culture where emotional distress is expressed through somatisation.

## Supplementary Information

**Additional file 1.**

## Data Availability

Data is available on request due to ethical restrictions imposed by Biomedical & Health Sciences, Dentistry, Medicine and Natural & Mathematical Sciences Research Ethics Subcommittees (BDM RESC) Kings College London. Requests for data access may be made to BDM RESC Kings College London through email bdm@kcl.ac.uk.
