# Supplementary material for: Detecting anxiety and depression among people with limited literacy living with chronic low back pain in Nigeria: adaptation and validation of the hospital anxiety and depression scale
Source: Arch Public Health. 2021 May 7;79:72. doi: 10.1186/s13690-021-00586-4 (PMC8105915; doi:10.1186/s13690-021-00586-4)
Supplement: Supplementary file 1 — Additional file 1. [file 13690_2021_586_MOESM1_ESM.docx]

## **Appendix**

**Igbo Hospital Anxiety and Depression Scale (Igbo-HADS)**

Ndi na afu ndi oria n’ulo ogwu maara na ihe na emetụta obi mmadu dị mkpa na ọtụtụ ọrịa. ọ bụrụ na onye na afu gị mara banyere ihe ndị a, ọ ga-enwe ike inyere gị aka nke oma.

Ajuju nchoputa a bu iji nyere onye na afu gi aka iji mara etu obi dị gị. A ga a gụru gi ihe ndi a, ma zakwaa nke biara gi na obi ngwangwa iji kowaa etu obi di gi **kemgbe izu uka gara aga**.

Egbuna oge iji zaa aziza ndi a, aziza i zara ozugbo bu ya nwere ike ikowa etu obi di gi karia aziza i chere eche oge tere aka wee zaa.

| D | Ihe ndi ahu na amasibu m ka na amasim |  |
| --- | --- | --- |
|  | Etu m si enwe ya na mbu kpom kwem | 0 |
|  | O buchaghi etu m si enwe na mbu | 1 |
|  | Nwantakiri | 2 |
|  | Oraka ahu | 3 |

| A | Onwe m adighi m juu: |  |
| --- | --- | --- |
|  | O foro ntakiri ka o buru mgbe nile | 3 |
|  | Otutu oge | 2 |
|  | Site n’oge ruo n’oge, kwa mgbe kwa mgbe | 1 |
|  | Adighi m enwe ya ma oli | 0 |

| A | A na m enwe ujo ka ihe di egwu o na akwado ime |  |
| --- | --- | --- |
|  | Kpomkwem ma dikwa njo nke ukwu | 3 |
|  | Ee, ma obughi na njo nke ukwu | 2 |
|  | Obere ma onaghi eche m uche | 1 |
|  | Odighi ma oli | 0 |

| D | E nwere m ike ichi ochi ma hukwa ihe itochi na uwa m |  |
| --- | --- | --- |
|  | Oke m nwere ike na mbu | 0 |
|  | O bughi etu odi na mbu ugbu a | 1 |
|  | Odighizi nnoo etu o di na mbu ugbu a | 2 |
|  | Odighi ma oli | 3 |

| A | Echiche nchekasi na aga na uche m |  |
| --- | --- | --- |
|  | Oge nke ukwuu | 3 |
|  | Otutu oge | 2 |
|  | Obughi kwa mgbe | 1 |
|  | Obere oge | 0 |

| D | A na m enwe onu |  |
| --- | --- | --- |
|  | Odighi ma oli | 3 |
|  | O bughi otutu oge | 2 |
|  | Mgbe ufodu | 1 |
|  | Otutu oge | 0 |

| D | A kwụsịrị m inwe mmasị n'etu m dị n'ile anya |  |
| --- | --- | --- |
|  | Kpom kwem | 3 |
|  | Anaghi m elebara ya anya dika m kwesiri | 2 |
|  | E nwere m ike i hapu ilebara ya anya dika o kwesiri | 1 |
|  | A na m elekota ya anya etu okwesiri | 0 |

| A | A na m enwe ujo dika ume mmiri a na efepu m na afo |  |
| --- | --- | --- |
|  | Odighi ma oli | 0 |
|  | Kwa mgbe kwa mgbe | 1 |
|  | Otutu mgbe | 2 |
|  | O foro ntakiri ka o buru mgbe nile | 3 |

| A | Adighi m enwe ezumike, enweghi m ike i no nwayo dika a ga asi na m gaghariba agari |  |
| --- | --- | --- |
|  | Nke ukwuu n’ezie | 3 |
|  | Otutu oge | 2 |
|  | Obughi nke ukwuu | 1 |
|  | Odighi ma oli | 0 |

| D | O di m ka a na m emezi ihe nwayo nwayo |  |
| --- | --- | --- |
|  | Oforo ihe nta ka o buru mgbe nile | 3 |
|  | Otutu mgbe | 2 |
|  | Mgbe ufodu | 1 |
|  | Odighi ma oli | 0 |

| A | E nwere m ike i no jii wee zuo ike |  |
| --- | --- | --- |
|  | Kpom kwe m | 0 |
|  | O na-emekari | 1 |
|  | O bughi otutu oge | 2 |
|  | Odighi ma oli | 3 |

| D | A na m ene anya i nwe anuri na ihe ga eme |  |
| --- | --- | --- |
|  | Ka m si eme na mbu | 0 |
|  | Etu na eruchaghi etu m si eme na mbu | 1 |
|  | Kpom kwem etu na erughi etu o di na mbu | 2 |
|  | Oraka ahu | 3 |

| A | Oke ujo na abia m na ike |  |
| --- | --- | --- |
|  | Otutu oge n’ezie | 3 |
|  | Otutu mgbe | 2 |
|  | Obuchaghi otutu oge | 1 |
|  | Odighi ma oli | 0 |

| D | A ga m enwenwu obi uto na igu akwukwo di mma ma o bu redio ma o bu ihe a na eme na TV |  |
| --- | --- | --- |
|  | Otutu mgbe | 0 |
|  | Mgbe ufodu | 1 |
|  | O bughi otutu oge | 2 |
|  | Oraka ahu | 3 |

**Original English Hospital Anxiety and Depression Scale (HADS)**

Doctors are aware that emotions play an important part in most illnesses. If your doctor knows about these feelings, he or she will be able to help you more. This questionnaire is designed to help your doctor know how you feel. Read each item and circle the reply which comes closest to how you have been feeling in the past week. Don’t take too long over your replies: your immediate reaction to each item will probably be more accurate than a long thought out response.

| D | I still enjoy the things I used to enjoy: |  |
| --- | --- | --- |
|  | Definitely as much | 0 |
|  | Not quite so much | 1 |
|  | Only a little | 2 |
|  | Hardly at all | 3 |

| A | I feel tense or ‘wound up’: |  |
| --- | --- | --- |
|  | Most of the time | 3 |
|  | A lot of the time | 2 |
|  | From time to time, occasionally | 1 |
|  | Not at all | 0 |

| A | I get a sort of frightened feeling as if something awful is about to happen: |  |
| --- | --- | --- |
|  | Very definitely and quite badly | 3 |
|  | Yes, but not too badly | 2 |
|  | A little, but it doesn’t worry me | 1 |
|  | Not at all | 0 |

| D | I can laugh and see the funny side of things: |  |
| --- | --- | --- |
|  | As much as I always could | 0 |
|  | Not quite so much now | 1 |
|  | Definitely not so much now | 2 |
|  | Not at all | 3 |

| D | I feel cheerful: |  |
| --- | --- | --- |
|  | Not at all | 3 |
|  | Not often | 2 |
|  | Sometimes | 1 |
|  | Most of the time | 0 |

| A | Worrying thoughts go through my mind: |  |
| --- | --- | --- |
|  | A great deal of the time | 3 |
|  | A lot of the time | 2 |
|  | Not too often | 1 |
|  | Very little | 0 |

| D | I have lost interest in my appearance: |  |
| --- | --- | --- |
|  | Definitely | 3 |
|  | I don’t take as much care as I should | 2 |
|  | I may not take quite as much care | 1 |
|  | I take just as much care as ever | 0 |

| A | I get a sort of frightened feeling like ‘butterflies’ in the stomach: |  |
| --- | --- | --- |
|  | Not at all | 0 |
|  | Occasionally | 1 |
|  | Quite often | 2 |
|  | Very often | 3 |

| D | I feel as if I am slowed down: |  |
| --- | --- | --- |
|  | Nearly all the time | 3 |
|  | Very often | 2 |
|  | Sometimes | 1 |
|  | Not at all | 0 |

| A | I get sudden feelings of panic: |  |
| --- | --- | --- |
|  | Very often indeed | 3 |
|  | Quite often | 2 |
|  | Not very often | 1 |
|  | Not at all | 0 |

| A | I feel restless as if I have to be on the move: |  |
| --- | --- | --- |
|  | Very much indeed | 3 |
|  | Quite a lot | 2 |
|  | Not very much | 1 |
|  | Not at all | 0 |

| D | I look forward with enjoyment to things: |  |
| --- | --- | --- |
|  | As much as I ever did | 0 |
|  | Rather less than I used to | 1 |
|  | Definitely less than I used to | 2 |
|  | Hardly at all | 3 |

| A | I can sit at ease and feel relaxed: |  |
| --- | --- | --- |
|  | Definitely | 0 |
|  | Usually | 1 |
|  | Not often | 2 |
|  | Not at all | 3 |

| D | I can enjoy a good book or radio or TV program: |  |
| --- | --- | --- |
|  | Often | 0 |
|  | Sometimes | 1 |
|  | Not often | 2 |
|  | Very seldom | 3 |
